# Glucose transporters in Alzheimer's disease

**DOI:** 10.1192/bjo.2021.707

**Published:** 2021-06-18

**Authors:** Natalia Kyrtata, Ben Dickie, Hedley Emsley, Laura Parkes

**Affiliations:** 1 The University Hospitals of Morecambe Bay Trust, The University of Manchester; 2 The University of Manchester; 3 Lancaster University

## Abstract

**Background:**

Physiological brain function depends on tight glucose regulation, including transport and phosphorylation, the first step in its metabolism. Impaired glucose regulation is increasingly implicated in the pathophysiology of Alzheimer's disease (AD). Glucose hypometabolism in AD may be at least partly due to impaired glucose transport at the blood-brain barrier (BBB). Glucose transporters (GLUTs) are an integral component of the BBB. There is evidence of a significant reduction in vascular and non-vascular forms of GLUT1 and GLUT3 in AD brains compared to age-matched controls. Glucose transport, as well as phosphorylation, appears to be a rate limiting step for glucose metabolism in the brain. We have reviewed the literature on glucose transport abnormalities in AD and the effect such abnormalities have on the brain.

**Method:**

Published literature between 1st January 1946 and 1st November 2019 was identified using EMBASE and MEDLINE databases and titles and abstracts were scanned. Human studies (autopsy and imaging) and data from animal models were included while reviews, letters and cellular or molecular studies were excluded from the search.

**Result:**

Autopsy studies in AD patients show significant reductions in GLUT3 in areas of the brain closely associated with AD pathology. Patients with AD and diabetes showed greater reductions of GLUT1 and GLUT3. A longitudinal study showed significant reductions in GLUT3 levels which correlated with greater amyloid-β (Aβ) and neurofibrillary tangle pathological burden in participants with AD pathology at post-mortem but without evidence of cognitive dysfunction in their lifetime. Some studies showed increased GLUT1, with others showing reduced GLUT1, levels in AD brain. A newly recognised GLUT12 appears to be increased in AD. Animal studies showed similar results with GLUT1 and GLUT3 knockout animal models exhibiting AD pathology, while overexpression of GLUT1 or treatment with metformin decreased Aβ toxicity in a Drosophila model of AD. GLUT2 levels were increased in both human AD brain and in an animal model of AD. Imaging studies using fluorodeoxyglucose [18F]FDG with positron emission tomography (FDG-PET) in AD subjects show reductions in glucose transport and glucose metabolism in areas most affected in AD. A small randomised control trial showed anti-diabetic medications improved the glucose transport in AD subjects.

**Conclusion:**

GLUTs play a significant role in AD pathology with evidence suggesting that GLUT3 reductions may precede the onset of clinical symptoms, while GLUT2 and GLUT12 may have a compensatory role. Repurposing anti-diabetic drugs shows promising results in both animal and human studies of AD.

